# Fc-Glycosylation in Human IgG1 and IgG3 Is Similar for Both Total and Anti-Red-Blood Cell Anti-K Antibodies

**DOI:** 10.3389/fimmu.2018.00129

**Published:** 2018-01-31

**Authors:** Myrthe E. Sonneveld, Carolien A. M. Koeleman, H. Rosina Plomp, Manfred Wuhrer, C. Ellen van der Schoot, Gestur Vidarsson

**Affiliations:** ^1^Department of Experimental Immunohematology, Sanquin Research, Amsterdam, Netherlands; ^2^Landsteiner Laboratory, Academic Medical Centre, University of Amsterdam, Amsterdam, Netherlands; ^3^Center for Proteomics and Metabolomics, Leiden University Medical Center, Leiden, Netherlands

**Keywords:** Fc glycosylation, IgG1, IgG3, IgG1 Fc, IgG3 Fc, antibodies, mass spectrometry

## Abstract

After albumin, immunoglobulin G (IgG) are the most abundant proteins in human serum, with IgG1 and IgG3 being the most abundant subclasses directed against protein antigens. The quality of the IgG-Fc-glycosylation has important functional consequences, which have been found to be skewed toward low fucosylation in some antigen-specific immune responses. This increases the affinity to IgG1-Fc-receptor (FcγR)IIIa/b and thereby directly affects downstream effector functions and disease severity. To date, antigen-specific IgG-glycosylation have not been analyzed for IgG3. Here, we analyzed 30 pregnant women with anti-K alloantibodies from a prospective screening cohort and compared the type of Fc-tail glycosylation of total serum- and antigen-specific IgG1 and IgG3 using mass spectrometry. Total serum IgG1 and IgG3 Fc-glycoprofiles were highly similar. Fc glycosylation of antigen-specific IgG varied greatly between individuals, but correlated significantly with each other for IgG1 and IgG3, except for bisection. However, although the magnitude of changes in fucosylation and galactosylation were similar for both subclasses, this was not the case for sialylation levels, which were significantly higher for both total and anti-K IgG3. We found that the combination of relative IgG1 and IgG3 Fc-glycosylation levels did not improve the prediction of anti-K mediated disease over IgG1 alone. In conclusion, Fc-glycosylation profiles of serum- and antigen-specific IgG1 and IgG3 are highly similar.

## Introduction

Immunoglobulins are the backbone of the adaptive humoral immune response generally formed to foreign antigens on pathogens and providing protection (1). However, we can also mount immune responses against foreign antigens found in other individuals of the same species (alloimmunization, frequently occurring during pregnancy or blood transfusion) or self-antigens, promoting allo- or autoimmune diseases, respectively. In cases of maternal immunization against red cells (RBC), only the immunoglobulin G (IgG) isotype can cross the placenta. These alloantibodies can cause destruction of RBCs in the fetus or newborn in reticuloendothelial cells in the spleen and/or Kupffer cells in the liver. This results in hemolytic disease of the fetus and newborn (HDNF), a potentially life-threatening disease (2). However, the potency of red cell antibodies to induce HDFN differs and depends on several factors including IgG subclass, specificity of the red cell antibodies, and level of expression on the fetal red cells and other tissues (2). HDFN caused by anti-D antibodies is well known, but may also occur due to other RBC antibody specificities, with anti-K, anti-c, and anti-E being most frequently implicated (3, 4). Severe HDFN, resulting in neonatal/fetal death or the need for a blood transfusion during pregnancy or within 1 week after delivery, most often occurs due to anti-D (33%), anti-K (26%), followed by anti-c (10%) and anti-E (2%) (2). Besides direct destruction of fetal red cells, anti-K antibodies can suppress erythropoiesis since—unlike the Rh antigens—K is also expressed on early RBC progenitors (5). Fetal anemia can lead to fetal hepatosplenomegaly as a result of compensatory erythropoiesis, hyperbilirubinemia due to fetal hemolysis, jaundice, kernicterus, or even fetal death (6).

Immunoglobulin G can be further divided into four subclasses: IgG1, IgG2, IgG3, and IgG4, which are produced in different amounts in response to antigens. Human IgG contains one highly conserved N-linked glycosylation site at position 297 which influences the interaction with FcγRs and C1q and thereby influence pro- and anti-inflammatory immune responses (7–13). This N-linked glycan consists of a biantennary glycan but varies widely in the exact structure. Each glycan on one of the two heavy chains carries zero, one or two galactoses, the majority contains a core fucose, a minority carries a bisecting *N*-acetylglucosamine (GlcNAc) (bisection), and one or two sialic acids may be attached on top of the galactoses (14). Together, this results in more than 20 glycan variants found on the IgG-Fc portion in human sera (15). Changes in total Fc-glycosylation, in particular decreased galactosylation and sialylation, have been described to contribute to a more inflammatory profile of the antibodies and have been described in various autoimmune diseases like rheumatoid arthritis, systemic lupus erythematosus, and inflammatory bowel disease (14, 16, 17). In addition, differences of IgG1 Fc-glycosylation of antigen-specific antibody subpopulations have been described compared to total IgG1 glycoprofiles. These changes are highly variable between individuals and are antigen dependent (9, 10, 12, 13, 18–21). In addition, marked changes of total or antigen-specific IgG1 fucosylation and galactosylation were found to correlate with clinical outcome of HDFN and fetal or neonatal alloimmune thrombocytopenia (FNAIT), making IgG1 glycosylation a possible screening marker for pathological IgGs (9, 10, 12, 13, 18). Similar changes for fucosylation have recently also been observed in immune response against HIV and dengue and have marked clinical consequences (19, 21).

In the studies described above, most work has been performed on the most abundant IgG subclass, IgG1 (7, 21–23), with only one study investigating glycan released from antigen-specific IgG of all subclasses (including potential Fab-glycans) (19). However, although most immune responses directed against protein antigens are of the IgG1 subclass, IgG3 responses can also dominate, especially early in the immune response, possibly because after immunoglobulin class-switching to IgG1 or IgG2-4, descendant cells can’t make IgG3 as the IgG3-heavy chain locus has then been excised from the genome (24). Theoretically, IgG3^+^ B-cells can however class switch further to IgG1 or to any other IgG class. Both indirect and direct experimental evidence for this has been obtain as somatic hypermutations seem to increase by subclass that mirrors the gene order (3 < 1 < 2 < 4) (25) and by sequencing of the resulting switch-region of IgG1 which can contain remnants of not only Sμ-Sγ1-derived sequences, but also Sγ3 (26). The tendency for this to occur is likely to depend on the antigen (e.g., T-independent antigens class switch directly to IgG2) (24), although one study has found remnants of Sγ3 sequences in 24% of IgG2 clones, indicating a serial-class switching from IgG3 to IgG2. 9% of IgG1 clones showed Sγ3-seqeunces in the same study (26). However, both IgG3 and IgG1 responses are frequently observed in responses against protein antigens, which is a common textbook knowledge for immune responses against many blood group antigens involved in alloimmune-mediated responses (27, 28).

This is important as the IgG subclasses differ in their ability to initiate phagocytosis *via* either complement or FcγR binding. IgG3 binds FcγRIII and C1q with higher affinity compared to IgG1 and is in some cases capable of mediating stronger effector functions *via* antibody-dependent cellular cytotoxicity (29, 30) and complement-dependent cytotoxicity (29, 31). As these functional features are affected by IgG-glycosylation (32), it is essential to study the relative glycosylation of the subclasses separately during these immune responses, and reveal possible glycosylation differences between subclasses. However, this is analytically challenging as the peptide backbone encompassing the N297-glycosylation site of IgG3, as released by trypsinization, is either identical with that of IgG2 or IgG4 depending on the IgG3 allotype (15, 24). By performing LC separation prior to mass spectrophotometry (MS) detection, proper distinction between IgG2 and IgG4 glycosylation can be performed (15, 33, 34). Previous studies have taken this into account and performed analyses on the comparison of IgG1 and collective IgG2/3 Fc-glycosylation patterns, showing galactosylation and sialylation profiles to be similar for both IgG1 and IgG2/3 during childhood and pregnancy (23, 35, 36). However, no study to date has reported antigen-specific IgG subclass glycosylation of more than one IgG subclass, with IgG3 being hitherto completely ignored (9, 10, 12, 13, 15, 19, 21, 37).

In the present study, we describe a simple and robust method for IgG3 N-glycan analysis. We compare Fc-glycosylation patterns of IgG1 and IgG3 for total- and antigen-specific IgG for alloimmune responses to Kell as a model for clinical evaluation of antibody responses where the IgG1-glycosylation profile has been shown to be predictive of disease severity. Immune reaction to this antigen is known to be restricted to IgG1 and IgG3 mostly without IgG2 and IgG4 (28) responses. Our hypothesis was that if the Fc-glycosylation profiles of IgG1 and IgG3 are not identical, then information on glycosylation of both subclasses may be required to predict clinical outcome in cases where both IgG1 and IgG3 responses are found.

## Materials and Methods

### Patient Samples

Patient samples from the prospective OPZI study (Detection and Prevention of Pregnancy Immunization) were included after informed consent (3). The OPZI study included all Dutch pregnant women with clinically relevant non-D RBC antibodies and antigen-negative children. Clinical data (hemoglobin levels and treatment with phototherapy or blood transfusion) were collected *via* healthcare workers as described before (3, 12). Clinical outcome was divided based on treatment given to the newborn in three groups: (i) severe (defined by an intrauterine or post-delivery blood transfusion given to the fetus or newborn, respectively), (ii) moderate (phototherapy was given to the newborn), or (iii) healthy (no treatment given to the newborn).

### Purification of Anti-K Antibodies from Sera

Anti-K alloantibodies from K-negative pregnant women were purified after incubation with K-positive RBCs as described before (12), and obtained after elution with a mild acid buffer. These women had earlier been diagnosed and confirmed with anti-K antibodies by Sanquin Diagnostics. In few cases, other antibodies than anti-K were identified, in which case anti-K purification was performed with K^+^ RBC but negative for the other antigen(s). The amount of IgG1 and IgG3 in the eluate and serum was determined by ELISA on 96-well plates (NUNC-Immuno, Maxisorp, Roskilde, Denmark) coated with either mouse-anti-human IgG1 Fc (clone M1325, Sanquin Reagents, Amsterdam, the Netherlands) or mouse-anti-human IgG3 hinge (clone M270, Sanquin Reagents). Fully human recombinant antibodies [IgG1κ and IgG3 GDob1 (38)] were used as standards to calculate the IgG1 and IgG3 concentrations. Eluates were considered to contain purified IgG1 and IgG3 when all negative controls were below a certain threshold.

### Purification of IgG1 from Sera and Antigen-Specific IgG1 and IgG3 from RBC Eluates

IgG1, IgG2/IgG3, and IgG4 (total IgG) were isolated from serum using 15 µL protein G Sepharose 4 Fast Flow beads (GE Healthcare Life Sciences, Uppsala, Sweden) as described earlier (9). Eluates from serum-opsonized K^+^ RBC containing anti-K specific IgG1 and IgG3 were purified as described previously by affinity chromatography, using 15 µL protein G Sepharose 4 Fast Flow beads (GE Healthcare Life Sciences, Uppsala, Sweden) in a 96-well plate (9). IgG1 and IgG3 glycopeptides were separated based on elution time and mass using liquid chromatography-mass spectrometry (LC-MS).

### Purification of IgG3 from Sera

IgG3 was isolated from serum in a 96-well sterile Multiscreen HTS plate (Milipore Corporation, Billerica, MA, USA). First, to remove IgG1, IgG2, and IgG4, 4 µL of serum was applied to 20 µL protein-A 4 Fast Flow beads (GE Healthcare Life Sciences, Uppsala, Sweden) in 150 µL of PBS, followed by a 1-h incubation at room temperature while shaking at 1,000 RPM (Heidolph Titramax 100, Namur, Belgium), using wells without protein-A beads as negative control. IgG3 was collected in the flow through after filtration. Next, 2.5 µL CaptureSelect™ IgG3 (Hu) affinity beads were added to the flow through and incubated for 1 h with shaking at 1,000 RPM (Heidolph Titramax 100, Namur, Belgium). IgG isolated from mixed control serum with protein G Sepharose 4 Fast Flow beads and PBS blanks without the addition of serum were used as controls. Plates were washed three times by filtration with 200 µL PBS, followed by three washing steps with 200 µL deionized water using a vacuum manifold. Bound IgG3 was eluted with 100 µL of 100 mM formic acid (Fluka, Steinheim, Germany) into a V-bottom microtitration plate (Nunc) using a vacuum manifold. Samples were dried in a vacuum concentrator at 45°C for 2.5 h.

### Mass Spectrometry Analysis of IgG1 and IgG3

The purified IgG1 and IgG3 were digested with initial shaking for 5 min followed by overnight incubation at 37°C with 20 µL 0.005 µg/µL trypsin (sequencing grade; Promega, Leiden, the Netherlands) in 25 mM ammonium bicarbonate. Samples were stored at −20°C until measurement. Analysis of purified tryptic IgG Fc-glycosylation was performed with nanoLC reversed-phase electrospray (ESI)-quadrupole time-of-flight (QTOF)-MS on an Ultimate 3000 RSLCnano system (Dionex/Thermo Scientific, Breda, the Netherlands) coupled to a Maxis Impact QTOF-MS (Bruker Daltonics, Bremen, Germany) as described previously (39).

Data processing was performed using LaCyTools (40). Separate analyte curation was performed for IgG1 and IgG3 for both serum and eluates. Tryptic IgG1 glycopeptides contained the peptide sequence EEQYNSTYR, while the peptide sequence for IgG3 varied according to allotype: the majority of the samples contained EEQFNSTFR, while two showed high signals for EEQFNSTYR (41). A list of *N*-glycopeptides were included in the LaCyTools analysis based on both characterization of the data and current knowledge of potential IgG glycans; *N*-glycopeptides were then excluded if either the average mass error of the 3+ charge state was below −10 or above 10 ppm, if the signal-to-noise ratio was above 9 or if the measure of isotopic pattern quality deviated over 0.35. A complete list of the *N*-glycopeptides which were included in the final analysis can be found in Table S1 in Supplementary Material. For each analyte, the area of the first x isotopic peaks was extracted for which the total signal amounted to at least 85%. A correction was then applied so that the values reflected 100% signal intensity. Signal intensities for various charge states were summed. The absolute intensities of the included analytes were normalized to the total area for IgG1 and IgG3 separately. For both IgG1 and IgG3, derived traits were calculated, showing the fraction of fucosylated, galactosylated and sialylated glycans, and glycans carrying a bisecting GlcNAc (the exact calculations are shown in Table S2 in Supplementary Material).

### Statistical Analysis

The derived traits as dependent variables tested not to be normally distributed using IBM SPSS 23.0 (IBM, Armonk, NY, USA). Linear regression was performed for correlating IgG1 to IgG3 Fc-glycosylation. Paired *t*-testing was performed comparing IgG1 and IgG3 Fc-glycosylation and Spearman rank to correlate Fc-glycosylation with Hb level. Fc-glycosylation was correlated to disease severity using one-way ANOVA testing with Bonferroni correction for multiple comparison. To further analyze the predicting value of both IgG1 and IgG3, we calculated the glycosylation of IgG1 and IgG3 combined as (relative anti-K IgG1 glycosylation) * ([IgG1 anti-K])/([IgG1 anti-K] + [IgG3 anti-K]) + (relative anti-K IgG3 glycosylation) * ([IgG3 anti-K])/([IgG1 anti-K] + [IgG3 anti-K]), where [IgG] is the concentration of the indicated subclass anti-K found in the RBC eluates. The reported *p*-values were considered statistically significant when below 0.05. The statistical tests were performed using SPSS 23.0 and GraphPad Prism version 6.00 for Windows (GraphPad Software Inc., La Jolla, CA, USA) and IBM SPSS Statistics for Windows version 23.0 (IBM Corp., Armonk, NY, USA).

## Results

### Antigen-Specific Anti-K Fc-Glycosylation Compared to Total IgG

**Figure 1:**
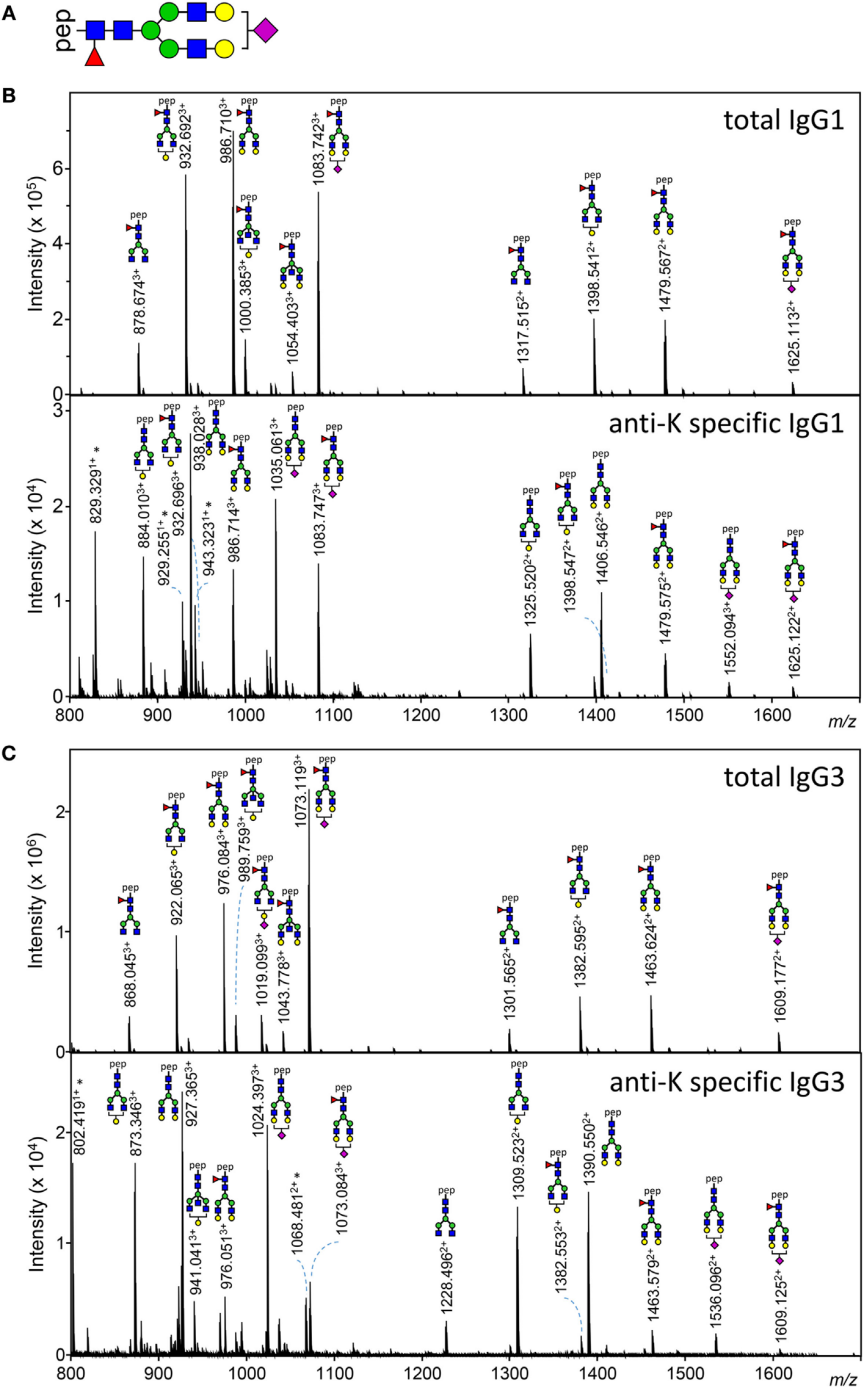
Examples of IgG1 and IgG3 Fc N-glycan structures showing skewing of anti-K Fc-glycosylation compared to total immunoglobulin G (IgG). **(A)** A schematic structure of the diantennary glycan found in human IgG. Blue square, *N*-acetylglucosamine; red triangle, fucose; green circle, mannose; yellow gray circle, galactose; purple diamond, *N*-acetylneuraminic (sialic) acid. **(B,C)** Mass spectrometric analysis of Fc glycopeptides encompassing N297 of IgG1 **(B)** and IgG3 **(C)**. Spectra from a representative patient are shown, with mass/charge ratio (*m/z*) of the glycopeptides on the *x*-axis and relative abundance on the *y*-axis. Asterisks indicate non-glycopeptide signals.

**Figure 2:**
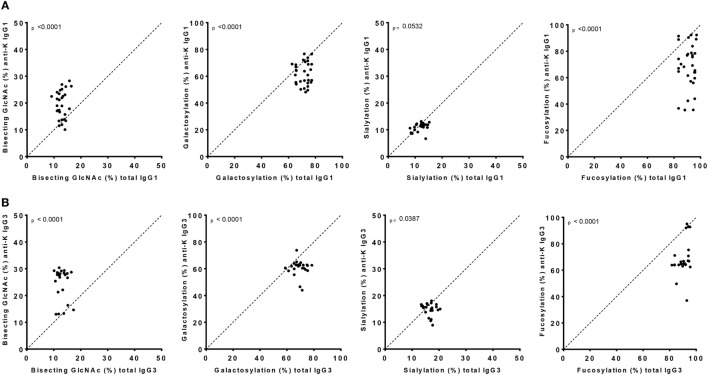
IgG1 and IgG3 antibodies show skewing of anti-K Fc-glycosylation compared to total immunoglobulin G (IgG). Glycosylation features found in alloantibodies against the Kell antigen. Total (*x*-axis) vs. antibody specific (*y*-axis) IgG1 **(A)** and IgG3 **(B)** shows a decreased anti-K fucosylation, galactosylation, and sialylation and increased bisection compared to total IgG. Statistical analysis was done using a paired *t*-test.

Both anti-K-specific and total serum IgG1 and IgG3 were purified from 30 pregnant women and the concentration of the purified anti-K IgG1 and IgG3 were quantified by ELISA (Table S3A in Supplementary Material). These eluates were digested with trypsin and the glycopeptides were subsequently analyzed by mass spectrometry. IgG1 Fc-glycosylation profiles were analyzed using a recently developed data processing tool employing additional quality assessment options (42) yielding results comparable to those obtained with a previous, less elaborate approach, yielding comparable results (12) (Figure 1). We then compared the antigen-specific glycosylation levels for IgG3 with the glycosylation level for total IgG3. Both total IgG3 and antigen-specific IgG3 were successfully obtained for 24 samples. Similar to IgG1-anti-K antibodies, IgG3-anti-K galactosylation, sialylation, and fucosylation were found to be decreased compared to total IgG3, while anti-K bisection was increased (Figure 2).

### Fc-Glycosylation of Total IgG3 and IgG1 Are Highly Similar within Each Individual

**Figure 3:**
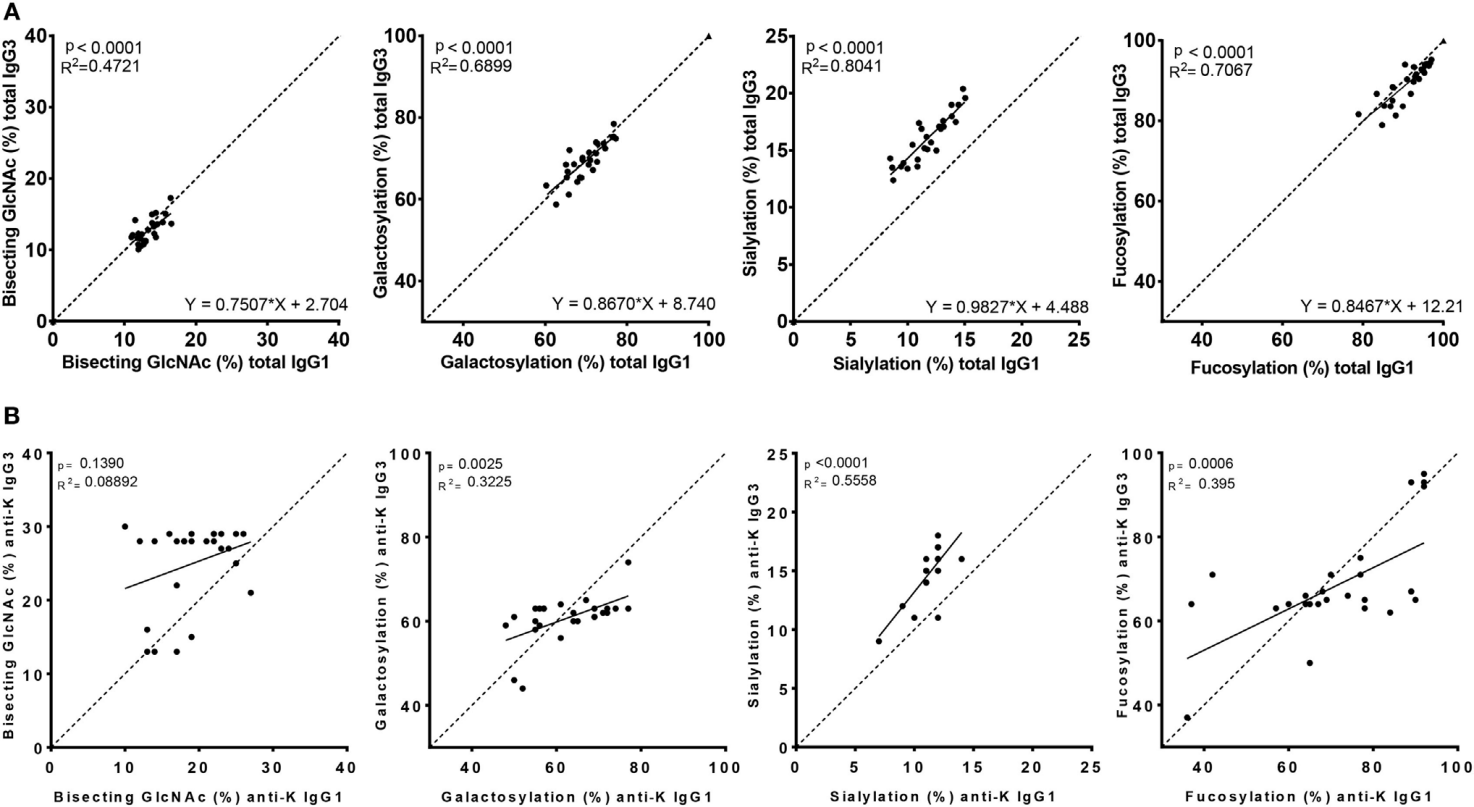
Correlation between IgG1 and IgG3 Fc-glycosylation for total and anti-K specific immunoglobulin G (IgG). Total **(A)** and anti-K **(B)** IgG1 (*x*-axis) vs. IgG3 (*y*-axis) Fc-glycosylation shows a significant correlation between fucosylation, bisecting GlcNAc, galactosylation, and sialylation except for anti-K bisecting GlcNAc. The dotted line indicates the line of identical IgG1 and IgG3 glycosylation and illustrates the similarity of total IgG1 and IgG3 glycosylation, except for sialylation which seems higher in IgG3. Statistical analysis was done using a linear regression analysis.

To analyze whether the glycosylation profiles of IgG1 and IgG3 were similar within an individual, we performed a linear regression analysis. A reliable IgG1 and IgG3 mass spectrometry result was obtained for 27 of these women. Glycosylation profiles of total IgG1 correlated significantly with those of total IgG3 for fucosylation, bisection, galactosylation, and sialylation (*p* < 0.0001) (Figure 3A).

However, the Fc-glycosylation profiles of total IgG1 and IgG3 were significantly different, except for galactosylation (71 vs. 70%) (Figure S1A in Supplementary Material). Quantitatively, these differences were only marginal for fucosylation and bisection (89.9 vs. 91.1%, *p* = 0.002 and 12.8 vs. 13.3%, *p* = 0.023, respectively, for IgG3 vs. IgG1), while sialylation was found to be much higher in IgG3 compared to IgG1 (16.3 vs. 11.8%, *p* < 0.0001).

### Antigen-Specific IgG-Glycosylation Responses Are Highly Analogous between IgG3 and IgG1

The Fc glycopeptides of purified antigen-specific anti-K IgG1 and IgG3 were also compared with each other (Figure 3B). The glycosylation profiles of anti-K specific IgG1 and IgG3 were also highly similar within individuals, which showed significant correlations between these subclasses for fucosylation, galactosylation, and sialylation, but not for bisection (Figure 3B). Fucosylation and galactosylation of antigen-specific anti-K antibodies was not significantly different between IgG1 and IgG3 (Figure S1B in Supplementary Material). Bisection was significantly increased for IgG3 compared to IgG1. In line with our findings for total serum IgG3, the most pronounced difference was found for sialylation, being higher in IgG3 compared to IgG1 (Figure S1B in Supplementary Material).

### Fc-Glycosylation and Antibody Levels of IgG1 and IgG3 Predict Clinical Outcome

**Figure 4:**
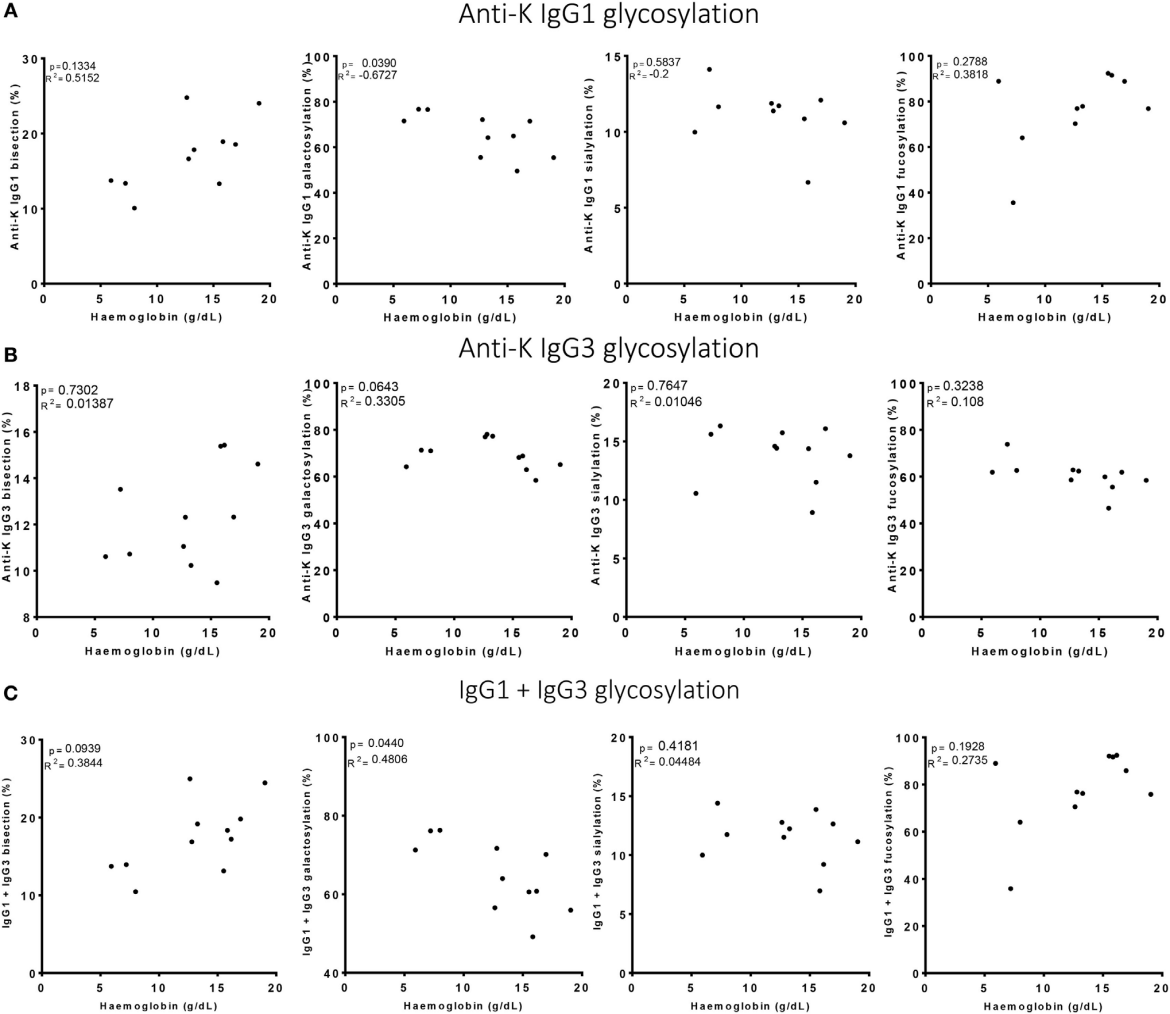
High galactosylation correlates with low Hb level. **(A)** Anti-K IgG1, **(B)** IgG3 glycosylation, and **(C)** the combination of IgG1 and IgG3 Fc-glycosylation and antibody level (*y*-axis) correlated to Hb level (x-axis). Galactosylation shows a significant correlation with Hb level for **(A)** anti-K IgG1 and **(C)** the combination of IgG1 and IgG3 Fc-glycosylation and antibody level. Statistical analysis was done using Spearman rank correlation.

**Figure 5:**
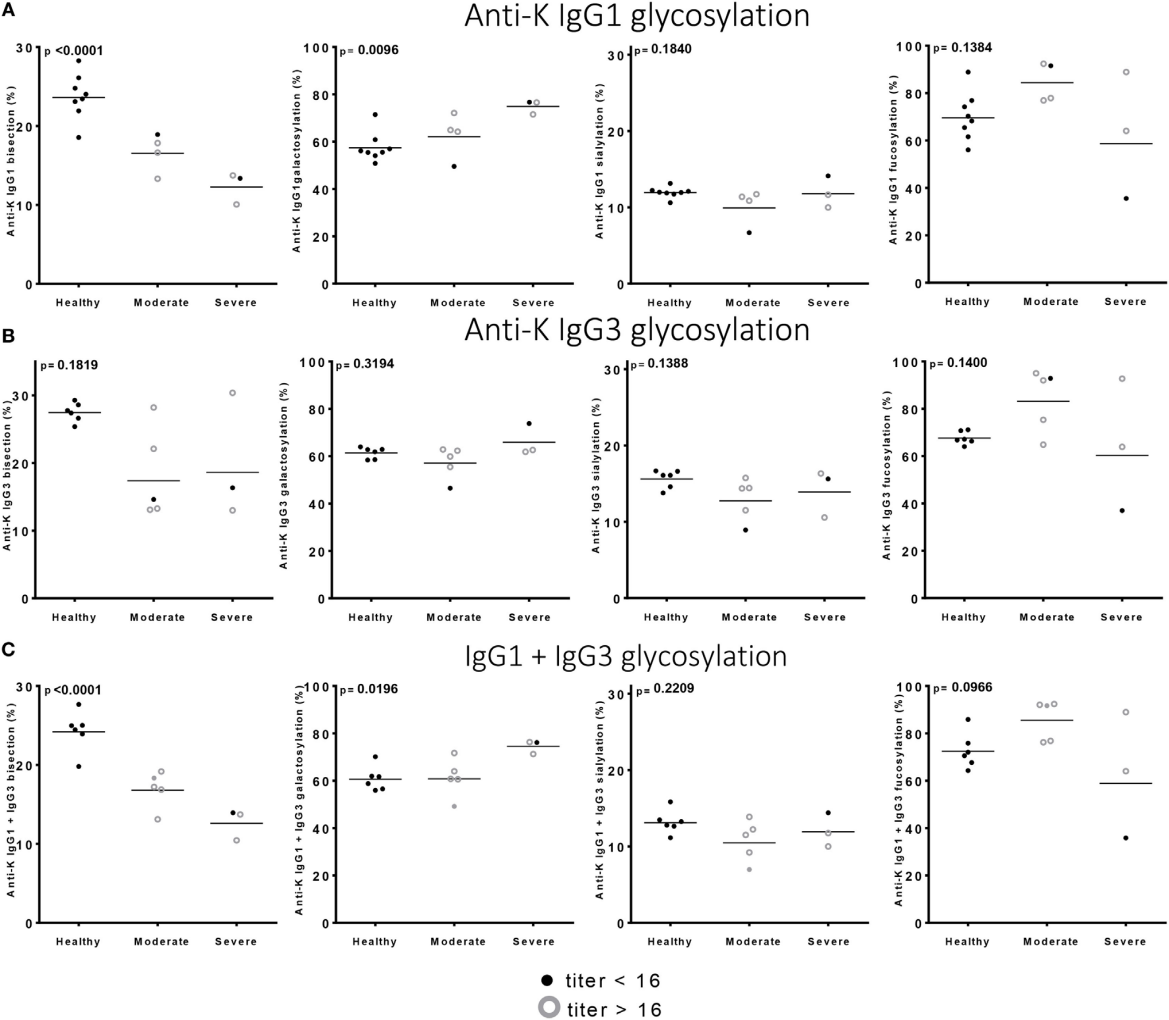
Low bisection and high galactosylation correlate with worse clinical outcome. **(A)** Anti-K IgG1, **(B)** IgG3 glycosylation, and **(C)** the combination of IgG1 and IgG3 Fc-glycosylation and antibody level (*y*-axis) correlated to disease severity (*x*-axis). Bisection and galactosylation show a significant correlation with disease severity. There is no correlation between fucosylation or sialylation and disease severity. Statistical analysis was done using one-way ANOVA with Bonferroni correction for multiple comparison testing.

Of the anti-Kell positive mothers, 17 gave birth to an antigen-positive child, making them at risk for HDFN (Table S3B in Supplementary Material). We attempted to improve the prediction of clinical outcome by combining the Fc-glycosylation levels of K-specific-IgG1 and IgG3 antibodies adjusted for the IgG1 and IgG3 concentration present (Table S3A in Supplementary Material). Due to the low sample size, a multiparametric analysis, such as stepwise forward multiple regression analysis including both titer and glycosylation levels, was not possible for meaningful analysis of these relationships with disease outcome, in this case hemolysis by anti-K in the affected fetuses. We therefore performed a Spearman rank correlation including Hb as outcome measurement, taking the glycosylation features of anti-K IgG1 (Figure 4A), IgG3 (Figure 4B), or weighted for the relative abundance of anti-Kell IgG1 and IgG3 present in the eluate (Figure 4C). Only the level of IgG1 galactosylation and the combined Fc-galactosylation of anti-Kell IgG1 and IgG3 level correlated in a comparable way with Hb level (Figures 4A,C). A borderline significant correlation was found for fucosylation and bisection, while no correlation was found for sialylation. However, based on the treatment given to the newborn, low fucosylation, low bisection, and high galactosylation did associate significantly with disease severity for IgG1 glycosylation alone and for the combined anti-Kell IgG1 and IgG3 Fc-glycosylation levels (Figures 5A,C) but not for IgG3 Fc-glycosylation alone (Figure 5B).

## Discussion

In this study, we analyzed Fc-glycosylation of IgG1 and IgG3 for both total serum and antigen-specific antibodies of 30 patient samples using mass spectrometry. We found that whereas the glycosylation profiles of both total and antigen-specific IgG1 and IgG3 are very similar within an individual, subtle differences exist, most pronounced for sialylation. In addition, we found that the relative levels of Fc-glycosylation for IgG1 and IgG3 combined only gave a marginally better prediction of clinical outcome compared to IgG1 Fc-glycosylation alone. Combined, this suggests that analysis of the dominant subclass in each individual (mostly IgG1) is sufficient to determine Fc glycosylation and assess its association with clinical parameters.

IgG3 glycosylation has not been extensively compared in normal individuals to that of IgG1 due to technological challenges, but like in this study similar glycosylation features to that of human IgG1 has been reported (15). The most prominent difference between the Fc glycan features of IgG1 and IgG2 we observed was the higher sialylation seen in IgG3. This may simply reflect different accessibilities of the Fc-N-glycan for different processing enzymes in the Golgi, due to differences in the quaternary structure of the Fc protein portions. An alternative explanation for the elevated IgG3 sialylation may also be that IgG3-Fc-sialylation is differently affected by either plasma sialidases or sialyltransferases (43) or increased upon the induction or boosting of immune responses, something that several studies have found both in mice and in humans (44, 45). As IgG3 is often made early against protein antigens but later disappear if they sequentially class switch to downstream subclasses or isotypes (24, 26), this may possibly explain the elevated sialylation in IgG3.

Although human IgG1-type antibody Fc-glycoprofiles have been studied in a handful of specific immune responses including infectious (14, 37, 44) and alloimmune diseases (9, 10, 12, 13), this is the first study that takes IgG3 into account and relates this to IgG1 glycosylation. Whereas IgG1 antibodies have almost exclusively high levels of core fucose, antigen-specific IgG1 antibodies in various alloimmune and some specific infectious diseases can display low levels of core fucosylation, although wide variation between different individuals is observed (10, 12, 13, 19, 21). Therefore, we hypothesized that a comprehensive study of alloantibodies against RBCs would enable us to investigate whether the production of low core fucosylated IgG occurs already at the IgG3 level or whether this glycoprofile is restricted to IgG1. Indeed, we found that the remarkable changes Fc fucosylation were similar for IgG1 and IgG3. In a previous study analyzing anti-K IgG1 Fc-glycosylation profiles of the same samples but using different data extraction techniques, we found similar trends, although the level of significance varied. The increased anti-K bisection was not observed in the previous study (12) but was detected in the current study using improved data processing and data curation methods (42).

We realize that our observation on specific immune responses is based only on alloantibodies against anti-K. Since similar trends were observed for total IgG, representing all IgG-responses within a given individual, this suggests the association between IgG1 and IgG3 glycosylation is a general phenomenon, but this has to be confirmed in other antigen-specific immune responses. The patients studied, formed the IgG response after transfusion or during pregnancy, with both immune responses giving similar responses in terms of glycosylation, including lowered core fucosylation (12). Similar responses were also observed in pregnancy and in voluntary immunized males against the RhD antigen (46). The pregnancy itself, the time of sampling, is also known to be a cause of elevated galactosylation and sialylation of IgG-Fc in general (10) that might theoretically mask any changes applied to antigen-specific IgG. Nevertheless, we have reported previously that the antigen-specific galactosylation—even in pregnancy—can be either increased or decreased compared to the total IgG galactosylation and sialylation. We now report that antigen-specific glycosylation features between IgG1 and IgG3 also strongly correlate within a given individual, despite this general tendency for temporal elevation in IgG galactosylation and sialylation during pregnancy.

The observed similarities in IgG1 and IgG3 glycosylation, both for total and in particular for antigen-specific responses, suggest that the regulation of glycosylation in B cells and plasma cells remains largely unaltered after class-switching from IgG3 to IgG1, at least for anti-K alloantibodies. If this can be confirmed for other immune responses, this suggests that the type of glycosylation machinery within a B cell may be determined at the moment of first encounter of the antigen and is largely retained during differentiation and expansion. The existence of an immunological memory for Fc-glycosylation is supported by our previous observations that the Fc-glycoprofiles of alloantibodies remain stable for over 7 years post-delivery (10), are maintained between pregnancies in FNAIT mothers (13) or during booster responses in hyperimmune anti-D donors (46). Together, this implies that glycosylation features of immunoglobulins are conserved after class-switching, either directly from an IgM-memory cell to IgG1 or IgG3, or from IgG3 to IgG1, and so are the altered functional properties of IgG1 and IgG3 inferred through these glycan changes (46).

Of the observed glycosylation differences, two are known to have effect on Fc-receptor or complement binding and subsequent functions. Fucose is known to be the most critical glycan component, with afucosylation inducing a strong increase in cytotoxicity of all IgG through elevated binding to FcγRIIIa and FcγRIIIb (10, 32, 47–49). Human IgG1 galactosylation has been implied to also enhance the effector function of IgG, with increased galactosylation enhancing binding to FcγRIIIa (8, 32) and C1q (11, 32), which is supported by clinical studies analyzing antigen-specific Fc-galactosylation (12, 13, 20, 37, 50). The impacts of these glycan changes on IgG3 functions are mostly unexplored. To our knowledge, the only documented cases is our recent study where we found that the effect of fucosylation on human FcγRIIIa/b binding is conserved for all human IgG subclasses (49), especially between IgG1 and IgG3. Given the extreme homologies between both FcγR- and C1q interactions between the IgG1 subclasses (24), the dependencies on interactions on glycan alterations is likely to be highly conserved, but still need verification.

Since IgG1 and IgG3 glycoprofiles correlated strongly with each other and are generally highly similar, one may hypothesize that glycoprofiles of either IgG1 or IgG3 would suffice to get a qualitative impression of the immune response. However, individual differences were noted, and it has been shown in other studies that in some cases, also seen here, that IgG1 and IgG3 can be found exclusively or predominantly (24). This is particularly important for analysis of clinically relevant anti-RBC antibodies, for example in pregnancy, when either or both IgG subclasses are frequently found. Here, we therefore analyzed both IgG1 and IgG3 Fc-glycosylation in relation to clinical outcome. In this study, only two women had far higher IgG3 levels than IgG1, with both women being sampled before giving birth to a Kell negative child, and are thus not clinically relevant. Since none of the maternal samples at risk for giving birth to a child with HDFN had a very high IgG3 concentration, the effect of IgG3 Fc-glycosylation was expected to be limited. Indeed, no additional value for predicting Hb level of the newborn was found using both relative IgG1 and IgG3 Fc-glycosylation levels as compared to IgG1 alone. Disease severity based on the treatment given to the newborn was most strongly associated with IgG1 galactosylation alone, but also with the combination of relative IgG1 and IgG3 galactosylation. These findings are in line with previously found associations between allo- and auto-RBC specific IgG1 glycosylation and Hb level as well as disease severity (12), and also with results for FNAIT (13). Low anti-RhD, anti-Kell and anti-HPA-1a IgG1 fucosylation and high galactosylation and sialylation of anti-Rhc and anti-auto-RBC IgG1 were found to correlate with more severe disease (9, 12).

Although in the current study the samples originated from a very large cohort including 679 pregnancies with anti-RBC antibodies, a possible limitation of this study is the small sample size and the western European (Dutch) origin of the subjects. Of the 679 included pregnancies, 230 were antigen positive (18 Kell, 117 Rhc, and 95 RhE) of which only 17 gave birth to a K-positive child, because the majority of the antibodies was provoked by a previous blood transfusion rather than by a K-positive child (12). Further analysis should be performed including a larger cohort, including non-pregnant subjects, of which clinical data are available.

In conclusion, this study shows that antigen-specific IgG1 and IgG3 responses in terms of their Fc-glycosylation are in general highly similar, with the exception of sialylation which was higher for IgG3 compared to IgG1. The remarkable reduction in core fucosylation seen previously in antigen-specific responses against both platelet and RBC antigens is also seen to a similar degree for IgG3. This indicates that the decision to produce low-fucosylated IgG is taken early in the immune response and that this information can be carried over by sequential class-switching from IgG3 producing cells to IgG1—which is known to occur (26)—showing signs of immunological memory as it is stable for years (10, 13, 46). Our previous work has indicated that glycosylation, especially fucosylation and galactosylation, were at least as important as antibody titer in determining clinical outcome (9, 12, 46), while others have shown that IgG1 and IgG3 have similar clinical activities (51). We found no improved prediction of the clinical outcome when including both IgG1 and IgG3 Fc-glycosylation levels, most likely because IgG3 concentrations are often low and associated glycosylation profiles are similar to those of IgG1. The danger of excluding IgG3 from analysis is that some cases have responses consisting almost exclusively of IgG3. However, the frequency of such cases is low, suggesting that monitoring IgG1 Fc-glycosylation profiles is sufficient for predicting disease severity in alloimmune-mediated diseases such as HDFN.

## Author Contributions

MS performed all purifications of anti-RBC alloantibodies from serum and isolated IgG subclasses; MS, CK, and HP conducted the mass spectrometry, processed the raw mass spectrometry data. MW and MS analyzed clinical data. MS, GV, and CS performed statistical analysis. MS, HP, CS, MW, and GV made figures and tables. CS, MW, and GV supervised the study. All authors contributed to analysis and interpretation of the data. MS, CS, MW, and GV wrote the paper, which was critically revised and approved by all authors.

## Conflict of Interest Statement

The authors declare that the research was conducted in the absence of any commercial or financial relationships that could be construed as a potential conflict of interest.
