# Supplementary material for: Fc-Glycosylation in Human IgG1 and IgG3 Is Similar for Both Total and Anti-Red-Blood Cell Anti-K Antibodies
Source: Front Immunol. 2018 Jan 31;9:129. doi: 10.3389/fimmu.2018.00129 (PMC5797742; doi:10.3389/fimmu.2018.00129)
Supplement: Supplementary file 1 [file Presentation_1.PPTX]

## Slide 1
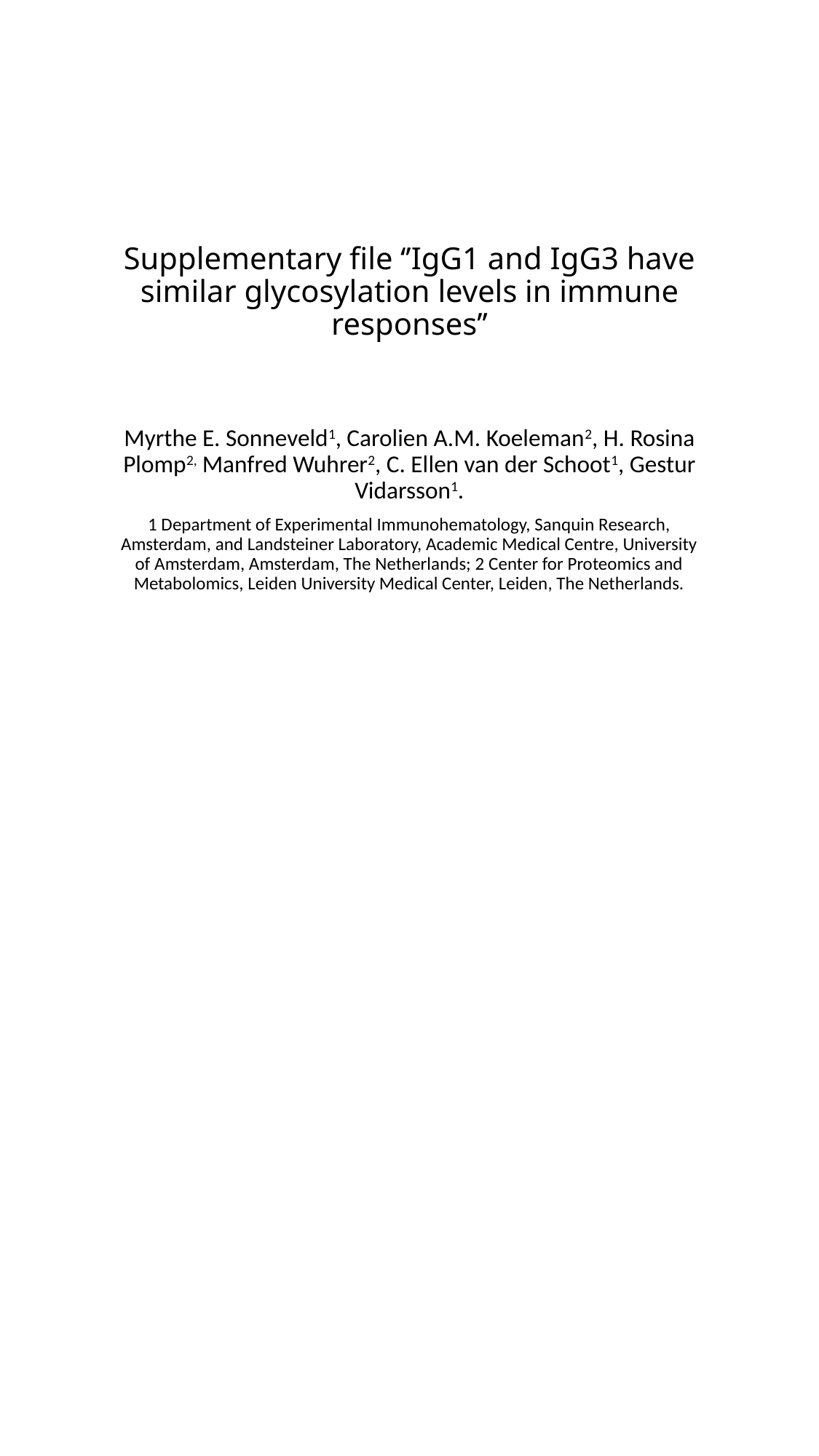

Supplementary file ‘’IgG1 and IgG3 have similar glycosylation levels in immune responses’’
Myrthe E. Sonneveld1, Carolien A.M. Koeleman2, H. Rosina Plomp2, Manfred Wuhrer2, C. Ellen van der Schoot1, Gestur Vidarsson1.
1 Department of Experimental Immunohematology, Sanquin Research, Amsterdam, and Landsteiner Laboratory, Academic Medical Centre, University of Amsterdam, Amsterdam, The Netherlands; 2 Center for Proteomics and Metabolomics, Leiden University Medical Center, Leiden, The Netherlands.

## Slide 2
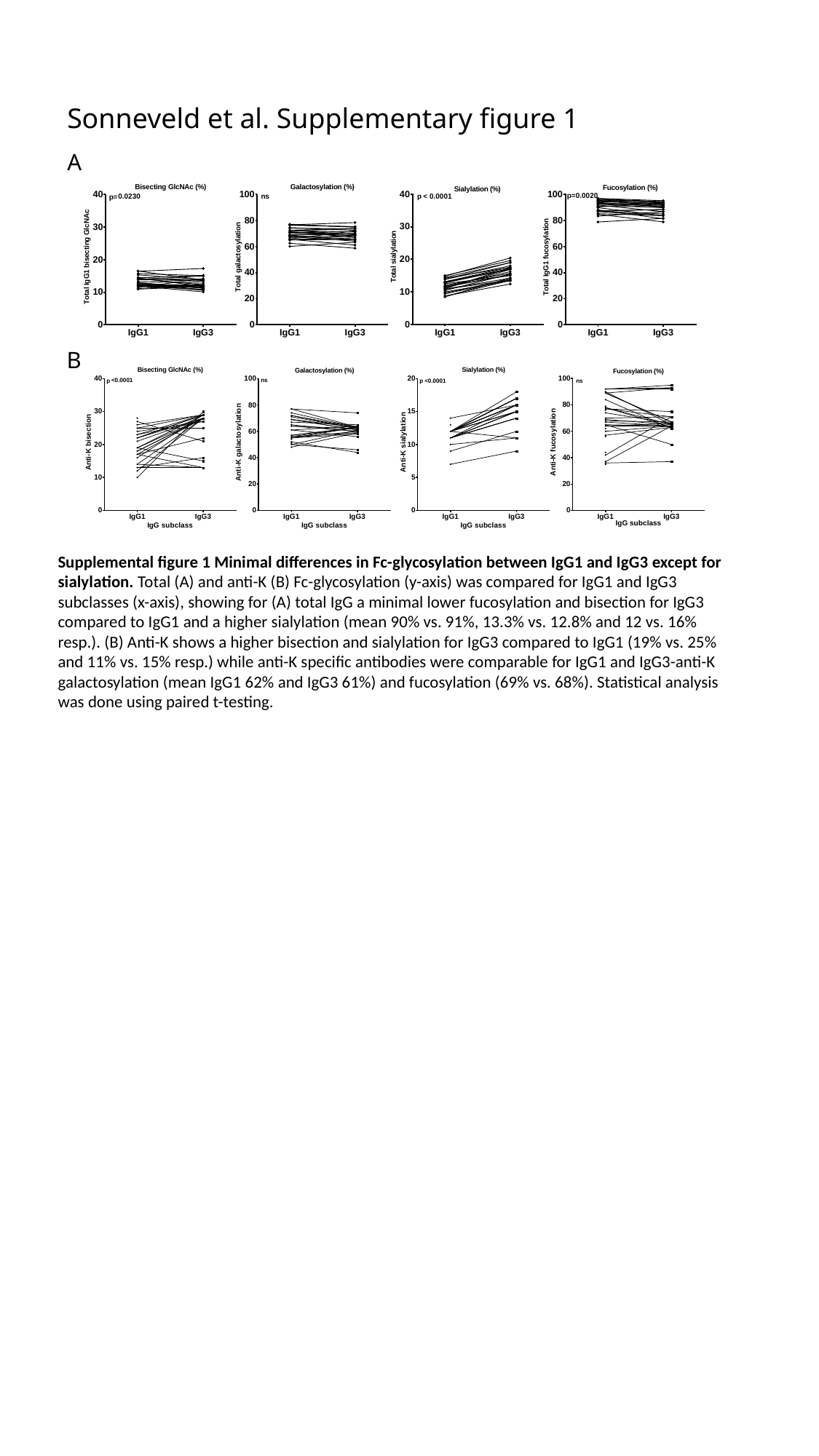

Sonneveld et al. Supplementary figure 1
A
B
Supplemental figure 1 Minimal differences in Fc-glycosylation between IgG1 and IgG3 except for sialylation. Total (A) and anti-K (B) Fc-glycosylation (y-axis) was compared for IgG1 and IgG3 subclasses (x-axis), showing for (A) total IgG a minimal lower fucosylation and bisection for IgG3 compared to IgG1 and a higher sialylation (mean 90% vs. 91%, 13.3% vs. 12.8% and 12 vs. 16% resp.). (B) Anti-K shows a higher bisection and sialylation for IgG3 compared to IgG1 (19% vs. 25% and 11% vs. 15% resp.) while anti-K specific antibodies were comparable for IgG1 and IgG3-anti-K galactosylation (mean IgG1 62% and IgG3 61%) and fucosylation (69% vs. 68%). Statistical analysis was done using paired t-testing.

## Slide 3
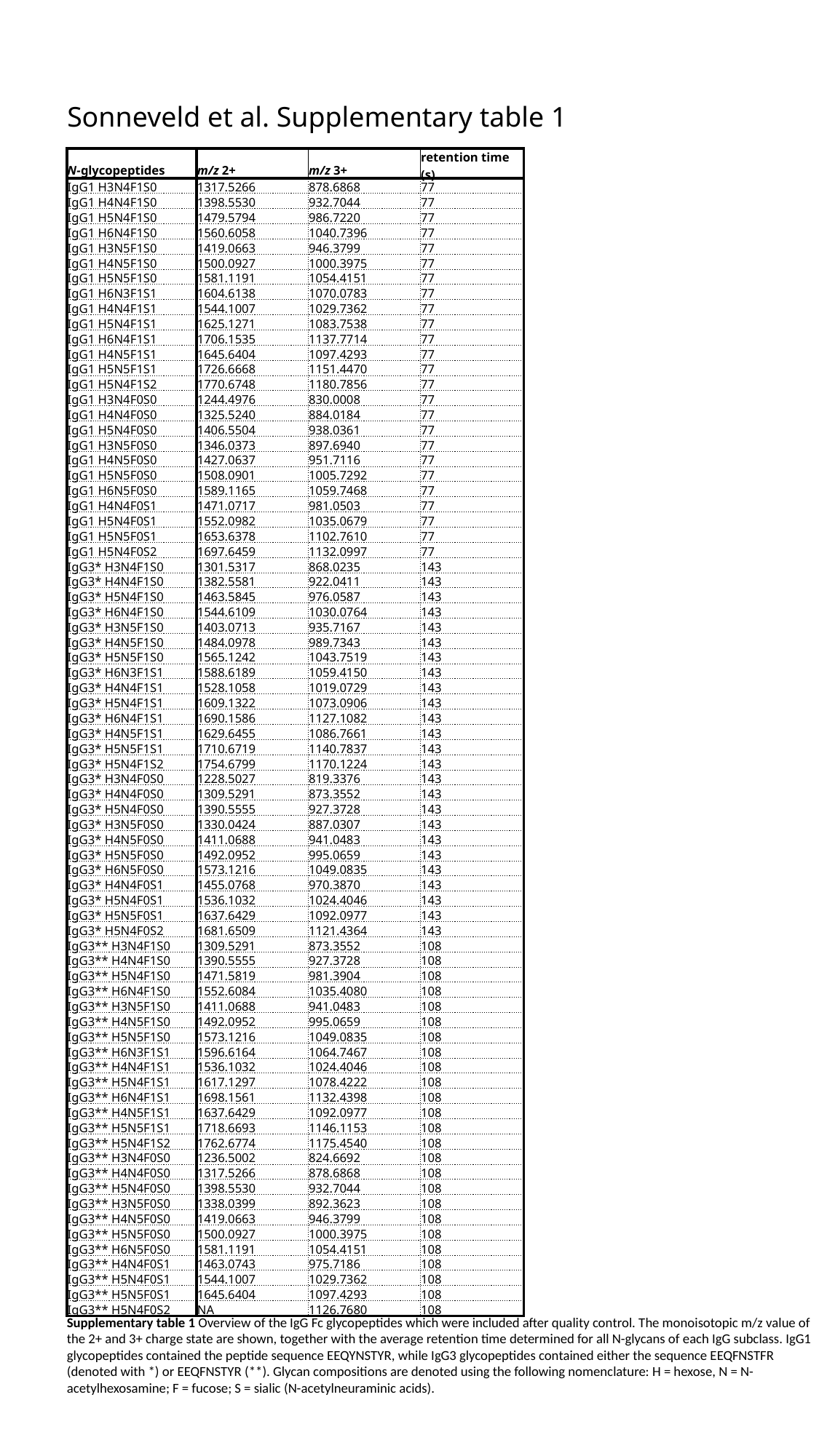

Sonneveld et al. Supplementary table 1
| N-glycopeptides | m/z 2+ | m/z 3+ | retention time (s) |
| --- | --- | --- | --- |
| IgG1 H3N4F1S0 | 1317.5266 | 878.6868 | 77 |
| IgG1 H4N4F1S0 | 1398.5530 | 932.7044 | 77 |
| IgG1 H5N4F1S0 | 1479.5794 | 986.7220 | 77 |
| IgG1 H6N4F1S0 | 1560.6058 | 1040.7396 | 77 |
| IgG1 H3N5F1S0 | 1419.0663 | 946.3799 | 77 |
| IgG1 H4N5F1S0 | 1500.0927 | 1000.3975 | 77 |
| IgG1 H5N5F1S0 | 1581.1191 | 1054.4151 | 77 |
| IgG1 H6N3F1S1 | 1604.6138 | 1070.0783 | 77 |
| IgG1 H4N4F1S1 | 1544.1007 | 1029.7362 | 77 |
| IgG1 H5N4F1S1 | 1625.1271 | 1083.7538 | 77 |
| IgG1 H6N4F1S1 | 1706.1535 | 1137.7714 | 77 |
| IgG1 H4N5F1S1 | 1645.6404 | 1097.4293 | 77 |
| IgG1 H5N5F1S1 | 1726.6668 | 1151.4470 | 77 |
| IgG1 H5N4F1S2 | 1770.6748 | 1180.7856 | 77 |
| IgG1 H3N4F0S0 | 1244.4976 | 830.0008 | 77 |
| IgG1 H4N4F0S0 | 1325.5240 | 884.0184 | 77 |
| IgG1 H5N4F0S0 | 1406.5504 | 938.0361 | 77 |
| IgG1 H3N5F0S0 | 1346.0373 | 897.6940 | 77 |
| IgG1 H4N5F0S0 | 1427.0637 | 951.7116 | 77 |
| IgG1 H5N5F0S0 | 1508.0901 | 1005.7292 | 77 |
| IgG1 H6N5F0S0 | 1589.1165 | 1059.7468 | 77 |
| IgG1 H4N4F0S1 | 1471.0717 | 981.0503 | 77 |
| IgG1 H5N4F0S1 | 1552.0982 | 1035.0679 | 77 |
| IgG1 H5N5F0S1 | 1653.6378 | 1102.7610 | 77 |
| IgG1 H5N4F0S2 | 1697.6459 | 1132.0997 | 77 |
| IgG3\* H3N4F1S0 | 1301.5317 | 868.0235 | 143 |
| IgG3\* H4N4F1S0 | 1382.5581 | 922.0411 | 143 |
| IgG3\* H5N4F1S0 | 1463.5845 | 976.0587 | 143 |
| IgG3\* H6N4F1S0 | 1544.6109 | 1030.0764 | 143 |
| IgG3\* H3N5F1S0 | 1403.0713 | 935.7167 | 143 |
| IgG3\* H4N5F1S0 | 1484.0978 | 989.7343 | 143 |
| IgG3\* H5N5F1S0 | 1565.1242 | 1043.7519 | 143 |
| IgG3\* H6N3F1S1 | 1588.6189 | 1059.4150 | 143 |
| IgG3\* H4N4F1S1 | 1528.1058 | 1019.0729 | 143 |
| IgG3\* H5N4F1S1 | 1609.1322 | 1073.0906 | 143 |
| IgG3\* H6N4F1S1 | 1690.1586 | 1127.1082 | 143 |
| IgG3\* H4N5F1S1 | 1629.6455 | 1086.7661 | 143 |
| IgG3\* H5N5F1S1 | 1710.6719 | 1140.7837 | 143 |
| IgG3\* H5N4F1S2 | 1754.6799 | 1170.1224 | 143 |
| IgG3\* H3N4F0S0 | 1228.5027 | 819.3376 | 143 |
| IgG3\* H4N4F0S0 | 1309.5291 | 873.3552 | 143 |
| IgG3\* H5N4F0S0 | 1390.5555 | 927.3728 | 143 |
| IgG3\* H3N5F0S0 | 1330.0424 | 887.0307 | 143 |
| IgG3\* H4N5F0S0 | 1411.0688 | 941.0483 | 143 |
| IgG3\* H5N5F0S0 | 1492.0952 | 995.0659 | 143 |
| IgG3\* H6N5F0S0 | 1573.1216 | 1049.0835 | 143 |
| IgG3\* H4N4F0S1 | 1455.0768 | 970.3870 | 143 |
| IgG3\* H5N4F0S1 | 1536.1032 | 1024.4046 | 143 |
| IgG3\* H5N5F0S1 | 1637.6429 | 1092.0977 | 143 |
| IgG3\* H5N4F0S2 | 1681.6509 | 1121.4364 | 143 |
| IgG3\*\* H3N4F1S0 | 1309.5291 | 873.3552 | 108 |
| IgG3\*\* H4N4F1S0 | 1390.5555 | 927.3728 | 108 |
| IgG3\*\* H5N4F1S0 | 1471.5819 | 981.3904 | 108 |
| IgG3\*\* H6N4F1S0 | 1552.6084 | 1035.4080 | 108 |
| IgG3\*\* H3N5F1S0 | 1411.0688 | 941.0483 | 108 |
| IgG3\*\* H4N5F1S0 | 1492.0952 | 995.0659 | 108 |
| IgG3\*\* H5N5F1S0 | 1573.1216 | 1049.0835 | 108 |
| IgG3\*\* H6N3F1S1 | 1596.6164 | 1064.7467 | 108 |
| IgG3\*\* H4N4F1S1 | 1536.1032 | 1024.4046 | 108 |
| IgG3\*\* H5N4F1S1 | 1617.1297 | 1078.4222 | 108 |
| IgG3\*\* H6N4F1S1 | 1698.1561 | 1132.4398 | 108 |
| IgG3\*\* H4N5F1S1 | 1637.6429 | 1092.0977 | 108 |
| IgG3\*\* H5N5F1S1 | 1718.6693 | 1146.1153 | 108 |
| IgG3\*\* H5N4F1S2 | 1762.6774 | 1175.4540 | 108 |
| IgG3\*\* H3N4F0S0 | 1236.5002 | 824.6692 | 108 |
| IgG3\*\* H4N4F0S0 | 1317.5266 | 878.6868 | 108 |
| IgG3\*\* H5N4F0S0 | 1398.5530 | 932.7044 | 108 |
| IgG3\*\* H3N5F0S0 | 1338.0399 | 892.3623 | 108 |
| IgG3\*\* H4N5F0S0 | 1419.0663 | 946.3799 | 108 |
| IgG3\*\* H5N5F0S0 | 1500.0927 | 1000.3975 | 108 |
| IgG3\*\* H6N5F0S0 | 1581.1191 | 1054.4151 | 108 |
| IgG3\*\* H4N4F0S1 | 1463.0743 | 975.7186 | 108 |
| IgG3\*\* H5N4F0S1 | 1544.1007 | 1029.7362 | 108 |
| IgG3\*\* H5N5F0S1 | 1645.6404 | 1097.4293 | 108 |
| IgG3\*\* H5N4F0S2 | NA | 1126.7680 | 108 |
Supplementary table 1 Overview of the IgG Fc glycopeptides which were included after quality control. The monoisotopic m/z value of the 2+ and 3+ charge state are shown, together with the average retention time determined for all N-glycans of each IgG subclass. IgG1 glycopeptides contained the peptide sequence EEQYNSTYR, while IgG3 glycopeptides contained either the sequence EEQFNSTFR (denoted with *) or EEQFNSTYR (**). Glycan compositions are denoted using the following nomenclature: H = hexose, N = N-acetylhexosamine; F = fucose; S = sialic (N-acetylneuraminic acids).

## Slide 4
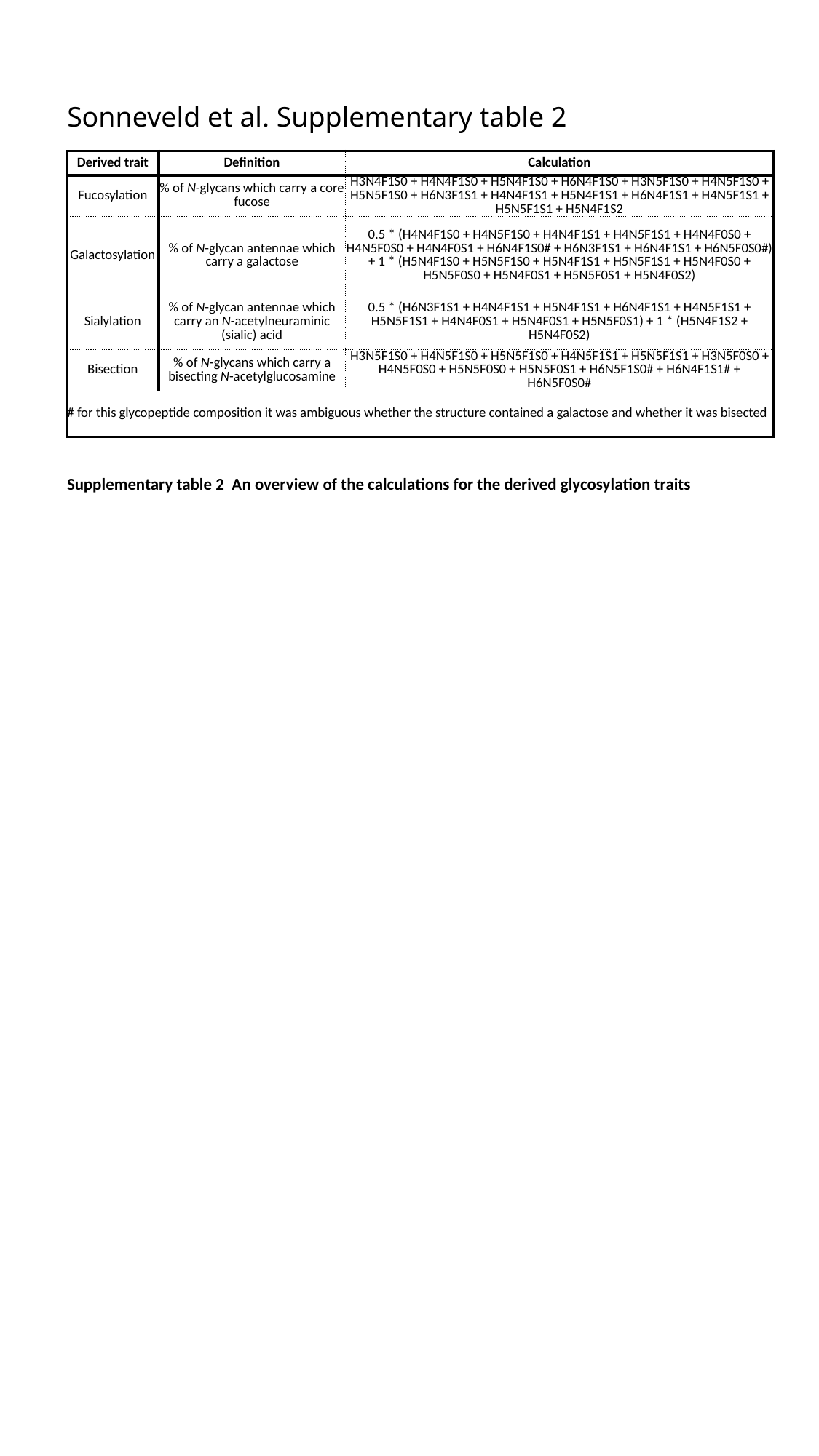

Sonneveld et al. Supplementary table 2
| Derived trait | Definition | Calculation |
| --- | --- | --- |
| Fucosylation | % of N-glycans which carry a core fucose | H3N4F1S0 + H4N4F1S0 + H5N4F1S0 + H6N4F1S0 + H3N5F1S0 + H4N5F1S0 + H5N5F1S0 + H6N3F1S1 + H4N4F1S1 + H5N4F1S1 + H6N4F1S1 + H4N5F1S1 + H5N5F1S1 + H5N4F1S2 |
| Galactosylation | % of N-glycan antennae which carry a galactose | 0.5 \* (H4N4F1S0 + H4N5F1S0 + H4N4F1S1 + H4N5F1S1 + H4N4F0S0 + H4N5F0S0 + H4N4F0S1 + H6N4F1S0# + H6N3F1S1 + H6N4F1S1 + H6N5F0S0#) + 1 \* (H5N4F1S0 + H5N5F1S0 + H5N4F1S1 + H5N5F1S1 + H5N4F0S0 + H5N5F0S0 + H5N4F0S1 + H5N5F0S1 + H5N4F0S2) |
| Sialylation | % of N-glycan antennae which carry an N-acetylneuraminic (sialic) acid | 0.5 \* (H6N3F1S1 + H4N4F1S1 + H5N4F1S1 + H6N4F1S1 + H4N5F1S1 + H5N5F1S1 + H4N4F0S1 + H5N4F0S1 + H5N5F0S1) + 1 \* (H5N4F1S2 + H5N4F0S2) |
| Bisection | % of N-glycans which carry a bisecting N-acetylglucosamine | H3N5F1S0 + H4N5F1S0 + H5N5F1S0 + H4N5F1S1 + H5N5F1S1 + H3N5F0S0 + H4N5F0S0 + H5N5F0S0 + H5N5F0S1 + H6N5F1S0# + H6N4F1S1# + H6N5F0S0# |
| # for this glycopeptide composition it was ambiguous whether the structure contained a galactose and whether it was bisected | | |
Supplementary table 2 An overview of the calculations for the derived glycosylation traits

## Slide 5
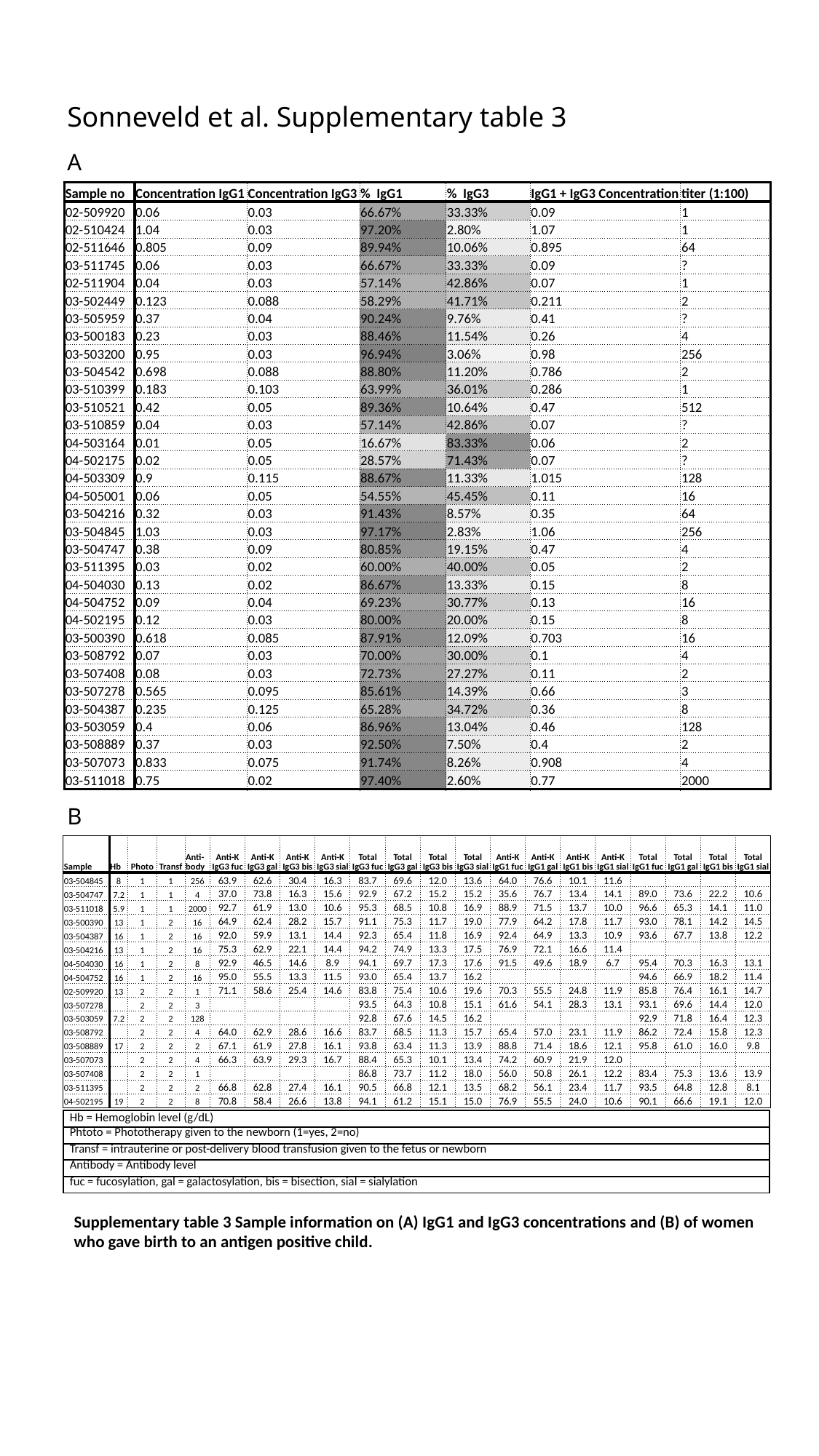

Sonneveld et al. Supplementary table 3
A
| Sample no | Concentration IgG1 | Concentration IgG3 | % IgG1 | % IgG3 | IgG1 + IgG3 Concentration | titer (1:100) |
| --- | --- | --- | --- | --- | --- | --- |
| 02-509920 | 0.06 | 0.03 | 66.67% | 33.33% | 0.09 | 1 |
| 02-510424 | 1.04 | 0.03 | 97.20% | 2.80% | 1.07 | 1 |
| 02-511646 | 0.805 | 0.09 | 89.94% | 10.06% | 0.895 | 64 |
| 03-511745 | 0.06 | 0.03 | 66.67% | 33.33% | 0.09 | ? |
| 02-511904 | 0.04 | 0.03 | 57.14% | 42.86% | 0.07 | 1 |
| 03-502449 | 0.123 | 0.088 | 58.29% | 41.71% | 0.211 | 2 |
| 03-505959 | 0.37 | 0.04 | 90.24% | 9.76% | 0.41 | ? |
| 03-500183 | 0.23 | 0.03 | 88.46% | 11.54% | 0.26 | 4 |
| 03-503200 | 0.95 | 0.03 | 96.94% | 3.06% | 0.98 | 256 |
| 03-504542 | 0.698 | 0.088 | 88.80% | 11.20% | 0.786 | 2 |
| 03-510399 | 0.183 | 0.103 | 63.99% | 36.01% | 0.286 | 1 |
| 03-510521 | 0.42 | 0.05 | 89.36% | 10.64% | 0.47 | 512 |
| 03-510859 | 0.04 | 0.03 | 57.14% | 42.86% | 0.07 | ? |
| 04-503164 | 0.01 | 0.05 | 16.67% | 83.33% | 0.06 | 2 |
| 04-502175 | 0.02 | 0.05 | 28.57% | 71.43% | 0.07 | ? |
| 04-503309 | 0.9 | 0.115 | 88.67% | 11.33% | 1.015 | 128 |
| 04-505001 | 0.06 | 0.05 | 54.55% | 45.45% | 0.11 | 16 |
| 03-504216 | 0.32 | 0.03 | 91.43% | 8.57% | 0.35 | 64 |
| 03-504845 | 1.03 | 0.03 | 97.17% | 2.83% | 1.06 | 256 |
| 03-504747 | 0.38 | 0.09 | 80.85% | 19.15% | 0.47 | 4 |
| 03-511395 | 0.03 | 0.02 | 60.00% | 40.00% | 0.05 | 2 |
| 04-504030 | 0.13 | 0.02 | 86.67% | 13.33% | 0.15 | 8 |
| 04-504752 | 0.09 | 0.04 | 69.23% | 30.77% | 0.13 | 16 |
| 04-502195 | 0.12 | 0.03 | 80.00% | 20.00% | 0.15 | 8 |
| 03-500390 | 0.618 | 0.085 | 87.91% | 12.09% | 0.703 | 16 |
| 03-508792 | 0.07 | 0.03 | 70.00% | 30.00% | 0.1 | 4 |
| 03-507408 | 0.08 | 0.03 | 72.73% | 27.27% | 0.11 | 2 |
| 03-507278 | 0.565 | 0.095 | 85.61% | 14.39% | 0.66 | 3 |
| 03-504387 | 0.235 | 0.125 | 65.28% | 34.72% | 0.36 | 8 |
| 03-503059 | 0.4 | 0.06 | 86.96% | 13.04% | 0.46 | 128 |
| 03-508889 | 0.37 | 0.03 | 92.50% | 7.50% | 0.4 | 2 |
| 03-507073 | 0.833 | 0.075 | 91.74% | 8.26% | 0.908 | 4 |
| 03-511018 | 0.75 | 0.02 | 97.40% | 2.60% | 0.77 | 2000 |
B
| Sample | Hb | Photo | Transf | Anti-body | Anti-K IgG3 fuc | Anti-K IgG3 gal | Anti-K IgG3 bis | Anti-K IgG3 sial | Total IgG3 fuc | Total IgG3 gal | Total IgG3 bis | Total IgG3 sial | Anti-K IgG1 fuc | Anti-K IgG1 gal | Anti-K IgG1 bis | Anti-K IgG1 sial | Total IgG1 fuc | Total IgG1 gal | Total IgG1 bis | Total IgG1 sial |
| --- | --- | --- | --- | --- | --- | --- | --- | --- | --- | --- | --- | --- | --- | --- | --- | --- | --- | --- | --- | --- |
| 03-504845 | 8 | 1 | 1 | 256 | 63.9 | 62.6 | 30.4 | 16.3 | 83.7 | 69.6 | 12.0 | 13.6 | 64.0 | 76.6 | 10.1 | 11.6 | | | | |
| 03-504747 | 7.2 | 1 | 1 | 4 | 37.0 | 73.8 | 16.3 | 15.6 | 92.9 | 67.2 | 15.2 | 15.2 | 35.6 | 76.7 | 13.4 | 14.1 | 89.0 | 73.6 | 22.2 | 10.6 |
| 03-511018 | 5.9 | 1 | 1 | 2000 | 92.7 | 61.9 | 13.0 | 10.6 | 95.3 | 68.5 | 10.8 | 16.9 | 88.9 | 71.5 | 13.7 | 10.0 | 96.6 | 65.3 | 14.1 | 11.0 |
| 03-500390 | 13 | 1 | 2 | 16 | 64.9 | 62.4 | 28.2 | 15.7 | 91.1 | 75.3 | 11.7 | 19.0 | 77.9 | 64.2 | 17.8 | 11.7 | 93.0 | 78.1 | 14.2 | 14.5 |
| 03-504387 | 16 | 1 | 2 | 16 | 92.0 | 59.9 | 13.1 | 14.4 | 92.3 | 65.4 | 11.8 | 16.9 | 92.4 | 64.9 | 13.3 | 10.9 | 93.6 | 67.7 | 13.8 | 12.2 |
| 03-504216 | 13 | 1 | 2 | 16 | 75.3 | 62.9 | 22.1 | 14.4 | 94.2 | 74.9 | 13.3 | 17.5 | 76.9 | 72.1 | 16.6 | 11.4 | | | | |
| 04-504030 | 16 | 1 | 2 | 8 | 92.9 | 46.5 | 14.6 | 8.9 | 94.1 | 69.7 | 17.3 | 17.6 | 91.5 | 49.6 | 18.9 | 6.7 | 95.4 | 70.3 | 16.3 | 13.1 |
| 04-504752 | 16 | 1 | 2 | 16 | 95.0 | 55.5 | 13.3 | 11.5 | 93.0 | 65.4 | 13.7 | 16.2 | | | | | 94.6 | 66.9 | 18.2 | 11.4 |
| 02-509920 | 13 | 2 | 2 | 1 | 71.1 | 58.6 | 25.4 | 14.6 | 83.8 | 75.4 | 10.6 | 19.6 | 70.3 | 55.5 | 24.8 | 11.9 | 85.8 | 76.4 | 16.1 | 14.7 |
| 03-507278 | | 2 | 2 | 3 | | | | | 93.5 | 64.3 | 10.8 | 15.1 | 61.6 | 54.1 | 28.3 | 13.1 | 93.1 | 69.6 | 14.4 | 12.0 |
| 03-503059 | 7.2 | 2 | 2 | 128 | | | | | 92.8 | 67.6 | 14.5 | 16.2 | | | | | 92.9 | 71.8 | 16.4 | 12.3 |
| 03-508792 | | 2 | 2 | 4 | 64.0 | 62.9 | 28.6 | 16.6 | 83.7 | 68.5 | 11.3 | 15.7 | 65.4 | 57.0 | 23.1 | 11.9 | 86.2 | 72.4 | 15.8 | 12.3 |
| 03-508889 | 17 | 2 | 2 | 2 | 67.1 | 61.9 | 27.8 | 16.1 | 93.8 | 63.4 | 11.3 | 13.9 | 88.8 | 71.4 | 18.6 | 12.1 | 95.8 | 61.0 | 16.0 | 9.8 |
| 03-507073 | | 2 | 2 | 4 | 66.3 | 63.9 | 29.3 | 16.7 | 88.4 | 65.3 | 10.1 | 13.4 | 74.2 | 60.9 | 21.9 | 12.0 | | | | |
| 03-507408 | | 2 | 2 | 1 | | | | | 86.8 | 73.7 | 11.2 | 18.0 | 56.0 | 50.8 | 26.1 | 12.2 | 83.4 | 75.3 | 13.6 | 13.9 |
| 03-511395 | | 2 | 2 | 2 | 66.8 | 62.8 | 27.4 | 16.1 | 90.5 | 66.8 | 12.1 | 13.5 | 68.2 | 56.1 | 23.4 | 11.7 | 93.5 | 64.8 | 12.8 | 8.1 |
| 04-502195 | 19 | 2 | 2 | 8 | 70.8 | 58.4 | 26.6 | 13.8 | 94.1 | 61.2 | 15.1 | 15.0 | 76.9 | 55.5 | 24.0 | 10.6 | 90.1 | 66.6 | 19.1 | 12.0 |
| Hb = Hemoglobin level (g/dL) |
| --- |
| Phtoto = Phototherapy given to the newborn (1=yes, 2=no) |
| Transf = intrauterine or post-delivery blood transfusion given to the fetus or newborn |
| Antibody = Antibody level |
| fuc = fucosylation, gal = galactosylation, bis = bisection, sial = sialylation |
Supplementary table 3 Sample information on (A) IgG1 and IgG3 concentrations and (B) of women who gave birth to an antigen positive child.
